# POLICY SERIES: OLDER AMERICANS ACT: REAUTHORIZATION PROCESS AND OUTCOMES

**DOI:** 10.1093/geroni/igz038.853

**Published:** 2019-11-08

**Authors:** Brian W Lindberg, Patricia M D’Antonio

**Affiliations:** The Gerontological Society of America, Washington, District of Columbia, United States

## Abstract

The Older Americans Act (OAA) reauthorization looks like the perfect bipartisan bill supporting older adults to move forward in 2019. This session provides an DC-insiders look at the process and outcomes, including analysis of proposed revisions to the law, and the roles of the House, Senate, and the Administration. Key players in the process make up the panel and will share their insights and predications on the Act’s future.

